# Intramuscular delivery of recombinant AAV expressing EpoR76E improves memory and is neuroprotective in 5xFAD transgenics

**DOI:** 10.21203/rs.3.rs-6465973/v1

**Published:** 2025-04-18

**Authors:** John Killmar, Yi Xue, Ruishan Wang, Tonia Rex, Mohammad Khan, Francesca-Fang Liao, Michael McDonald

**Affiliations:** University of Tennessee Health Science Center

**Keywords:** Gene therapy, erythropoietin, cognition, memory, neurotrophic factors, Alzheimer’s disease, neuroprotection

## Abstract

Converging evidence suggests that erythropoietin (Epo) may be effective in alleviating symptoms of many neurological conditions, including traumatic brain injury and neurodegenerative disorders. However, a limitation to its use as a therapeutic agent is the risk associated with stimulation of hematopoietic pathways. To overcome this issue, we used a recombinant adeno-associated viral vector (AAV) designed to express a modified form of erythropoietin devoid of hematopoietic activity, EpoR76E. Our previous research showed that AAV.EpoR76E prevented motor impairments and mitigated loss of dopaminergic neurons in the MPTP mouse model of Parkinson’s disease. In the present study, a single intramuscular injection of AAV expressing EpoR76E prevented cognitive decline in the 5xFAD transgenic model of Alzheimer’s disease. Consistent with this, AAV-EpoR76E prevented the age-related loss of pre-and post-synaptic proteins synaptophysin and PSD-95 normally seen in 5xFAD transgenics. Additionally, the treatment reduced soluble and aggregated amyloid-β levels in 5xFAD mice, and prevented the loss of neurons in the medial septum and vertical limb of the diagonal band, the primary cholinergic projections to the hippocampus. Together, these results suggest that AAV-EpoR76E might represent a novel therapeutic approach for Alzheimer’s disease and other neurodegenerative disorders.

## Introduction

Considered the most prevalent dementing illness worldwide, Alzheimer’s disease is characterized by the intracellular aggregation of hyperphosphorylated tau protein and the extracellular deposition of amyloid plaques in the brain [1, 2]. While the events that initiate the disease process is not known, it is widely accepted that temporal accumulation of these two neuropathological hallmarks leads to neurodegeneration, neuroinflammation, and synaptic pathology as well as clinical symptoms including impaired memory, attention, and language skills [3–5].

The primary function of the glycoprotein erythropoietin (Epo) is the production and regulation of red blood cells through dedicated homodimeric receptors [6]. However, it has long been known that exogenous Epo also has neuroprotective properties in multiple neurological conditions [7–9]. In preclinical models of traumatic brain injury, ischemia/hypoxia, or neurodegeneration, Epo-mediated treatments promote neuroprotection, improve memory, reduce tau pathology, and stimulate hippocampal neurogenesis [10–16]. This suggests that Epo may represent a plausible neuroprotective agent [17, 18]. One limitation of systemic Epo therapy, however, is the risk of concomitant stimulation of hematopoiesis, possibly resulting in increased hematocrit and potentially serious clinical complications.

Efforts to develop Epo variants for Alzheimer’s disease that do not increase hematocrit have had some success [18–21]. A recent clinical trial of subjects 50 years and older exhibiting mild-to-moderate Alzheimer’s symptoms showed significant improvement in clinical cognitive tests over the 48-week treatment period with thrice-weekly intranasal administration of a non-erythropoietic hyposialylated Epo [22]. Cognition worsened significantly in the placebo group. They did not examine pathological variables in that study, but there was an equivalent decrease in hippocampal volume in subjects receiving placebo or either dose of experimental Epo.

A gene therapy approach involving EpoR76E, an Epo variant with attenuated hematopoietic activity, has had considerable preclinical success [23–30]. In addition to its ability to attenuate symptoms ocular injury and disease, a single intramuscular injection of an adeno-associated viral vector (AAV) expressing EpoR76E was sufficient to prevent parkinsonism and promote the survival of dopaminergic neurons in mice injected with 1-methy-4-phenyl-1,2,3,6-tetrahydropyridine (MPTP), a model of Parkinson’s disease [31]. The long-lasting effects of AAV-driven EpoR76E make it an ideal candidate for potential treatment of age-related neurodegenerative diseases. In the present study, we sought to determine whether a singular intramuscular injection of AAV.EpoR76E would attenuate neuropathology and memory deficits in the 5xFAD model of Alzheimer’s disease. These transgenics exhibit robust cognitive impairments and amyloid-β (Aβ) aggregation as early as 4 months of age [32, 33] and neuronal loss in cortical layer V, the subiculum, and medial septal area by 9 months of age [34, 35], enabling us to evaluate neurodegeneration as well as the other hallmark features of Alzheimer’s disease.

## Material & Methods

### Subjects.

Subjects were all bred from male 5xFAD transgenic mice from Jackson Laboratory (stock #006554) and female non-Tg controls (strain B6SJLF1/J; stock #100012). The transgenic mice harbor three point mutations (I716V, V717I, & KM670/671NL) in a human amyloid precursor protein (APP) transgene and two point mutations (M146L & L286V) in a human presenilin 1 (PSEN1) transgene, which segregate together in these mice. All experimental mice were bred in-house and were hemizygous for both mutant transgenes. PCR analyses of genomic DNA from tail biopsies were performed before weaning on all mice to confirm APP and PSEN1 genotypes. Mice homozygous for either *Pde6b*^*rd1*^ or *Dysf*^*im*^ mutant alleles (leading to retinal degeneration & spontaneous peripheral myopathy, respectively), which segregate in the background strain, were identified and excluded from behavioral testing. Mice were group-housed in standard cages (29 × 19 × 13 cm) with wood-shaving bedding in an AAALAC-accredited facility (temperature- and humidity-controlled room, 12 h light/dark cycle, lights on at 6 am). Behavioral assessments were conducted during the light phase of the cycle. Food and water were available *ad libitum* except during DNMTP testing as described below; during this time subjects had free access to food for 3 hours per day. This resulted in a moderate weight loss to 85 to 90% of their free-feeding weights initially followed by weight gain for the duration of the study despite continued food restriction. All procedures were approved by the Institutional Animal Care and Use Committee at the University of Tennessee Health Science Center.

### Generation of AAV vectors.

EpoR76E was generated by site-directed mutagenesis of the erythropoietin gene (EPO) of rhesus macaque, substituting a glutamate for arginine at position 76 [23, 30, 31, 36]. The recombinant AAV vectors were produced by the University of Iowa Vector Core as previously described [30, 37–41]. The AAV2/5.CMV.EpoR76E and AAV2/5.CMV.eGFP (negative control) vectors were isolated and purified from the cells by double cesium chloride gradient centrifugation and dialyzed in HEPES-buffered saline, pH 7.8 [42]. The titers were determined by a quantitative real-time polymerase chain reaction (qPCR) assay using primers specific to the corresponding poly-A tail.

### Viral vector administration.

Three days after the challenge DNMTP sessions (see below), 5.5–6 month-old mice were anesthetized under isoflurane and given a single injection of AAV.EpoR76E or AAV.eGFP in the gastrocnemius muscle. The titer of AAV.EpoR76E was 5.0 × 10^10^ GC/mL, justified by a pilot study showing that a higher titer (1.0 × 10^11^ GC/mL) led to increased hematocrit levels in B6SJL/J mice for 3 weeks before returning to normal. The higher titer did not increase hematocrit in younger, wild-type C57BL/6N mice [31]. Approximately equivalent numbers of male and female mice were assigned to each experimental condition.

### Cell culture.

The Chinese hamster ovary cell line 7PA2 was used to examine the effects of Epo on Aβ secretion *in vitro*. 7PA2 cells stably express the Val717Phe familial APP mutation and are the most characterized cellular model of naturally-secreted Aβ [43]. Cells were cultured in Dulbecco’s Modified Eagle Medium with 10% fetal bovine serum, 2mM L-glutamine, 1% penicillin/streptomycin, and 200 μg/ml G418 (Invitrogen). The human Epo coding sequence from plenti6.3-hEPO (Addgene, #50436) was subcloned into a pcDNA3.1(+) vector with an HA tag at the c-terminal, and mutants were generated with QuikChange II Site-Directed Mutagenesis Kit (Agilent). The primers used were hEPO_S71E F: 5’ ctggccctgctgGAggaagctg 3’, hEPO_S71E R: 5’ cagcttccTCcagcagggccag 3’, hEPO_R76E F: 5’ gctgtcctgGAgggccaggccct 3’, hEPO_R76E R: 5’ agggcctggcccTCcaggacagc 3’, hEPO_s71E/R76E F: 5’ gccctgctgGAggaagctgtcctgGAgggccaggcc 3’, and hEPO_s71E/R76E R: 5’ ggcctggcccTCcaggacagcttccTCcagcagggc 3’. Epo and its mutants were transfected into 7PA2 cells using lipofectamine 2000 (Invitrogen) with transfection of the empty vector as a control. Twenty-four hours after transfection, cells were incubated in FBS-free medium overnight. Secreted proteins were precipitated with trichloroacetic acid and analyzed by immunoblotting as previously described [44]. Antibodies used are listed in Table 1. Epo and its variants were detected using an antibody to the HA tag contained in the plasmids.

Sensorimotor function, anxiety, and cognition were investigated beginning at 9 months of age. Mice were successively tested on the rotarod, horizontal beam, grid suspension, light/dark exploration, elevated plus maze, and cross maze tests. Except for the first three tests, all procedures were videorecorded using AnyMaze software (Stoelting, USA).

### Sensorimotor function.

Balance and motor coordination were assessed in a rotarod (Rotamex-5; Columbus Instruments, USA). Animals were trained for 6 trials per day over a 4-day period on a rotating rod (3 cm diameter). Rotation speed ranged from 0 to 48 rpm over a 6-min trial, with acceleration of 2 rpm every 15 s. Each daily session started with a practice trial. For all subsequent trials, the latency to fall from the rod (directly onto a soft surface placed beneath or by rotating directly around the spindle) was recorded. For short (< 30 s) fall latencies, mice were immediately given a second trial, this second measurement providing the final score for that trial regardless of its duration. Motor balance was measured in the horizontal beam task [31, 45, 46]. Briefly, mice were trained to cross an 81-cm-long beam in order to escape a brightly-lit 5-cm^2^ platform and reach a dark, “safe” compartment (23 × 20 × 18 cm). The 1-cm round beam was elevated 40 cm above soft blankets in case of a fall. Mice were given a total of 3 trials, during which the latency to traverse the entire beam measured. All trials were video-recorded with a digital camera (Panasonic, USA) to score the number of foot-slips. In the grid suspension test, mice were placed on a metal wire grid (24 X 19 cm) that was turned upside down, approximately 40 cm above soft foam cushions. Once the grid was fully inverted, the average latency to fall was measured for each animal over three 60-s trials, with 1-min ITIs between trials. Each mouse was also given an activity score to rate its movement on the grid, from 0 (inactive) to 3 (very active). Grip strength was assessed using a commercially-available grip-strength meter (Columbus Instruments, Colombus, OH) as previously described [45]. Strength of forepaw grip was measured followed by strength of all four paws, for three trials each. On each trial, the subject was lowered slowly to a screen from above by the base of the tail. Once the mouse gripped the screen the experimenter gently pulled the mouse away from the apparatus with consistent force until it let go. The meter automatically recorded the maximal force.

#### Anxiety-related behaviors.

The light/dark and elevated plus maze tests are commonly-used procedures to evaluate anxiety in rodents as previously described in our lab [47, 48] [35] [49]. Mice were allowed to rest undisturbed for 90 min. following transportation from the vivarium to the testing rooms. The light/dark task was conducted in a white acrylic arena (50 × 25 × 22 cm) fitted with a black acrylic insert (25 × 25 × 22 cm) placed inside to split the total area into two compartments of similar size. A small opening (5 × 5 cm) enabled the subject to circulate back and forth between light and dark compartments. Each mouse was introduced in the lighted area and allowed to explore freely over a 5-min session. The cumulative amount of time spent in both areas and the total number of transitions were quantified. Following that, mice were evaluated in a 40-cm elevated plus maze apparatus consisting of two open arms (30 × 6 cm, no walls) and two closed arms (30 × 6 cm, 15-cm high walls) connected by a central area (6 × 6 cm). To begin a session, a mouse was introduced in the central part of the maze and allowed to explore for 5 min. The percentage of time spent on closed vs. open arms (excluding time spent in the center), and the number of entries into closed and open arms were measures of interest. Non-transgenic mice usually spend more time in secure (closed) arms; however, 5xFAD mice spend more time in aversive (open) arms, presumably due to the loss of layer IV barrel field inhibitory interneurons. Transgenics without whiskers perform normally on the plus maze and all other anxiety tests [35].

### Cross-maze spontaneous alternation.

Spatial memory was measured using cross-maze spontaneous alternation. We and others have shown that 5xFAD transgenics exhibit a robust impairment on this task. The cross maze was a plus-shaped, white acrylic apparatus comprising four identical arms (30 × 5 × 15 cm) and a central area (5 × 5 cm). At the beginning of the session a mouse was placed at the end of an arm. The same starting point was used for every subject. During the 10-min session, the experimenter recorded sequential visits to the arms (designated A through D). Alternation rate, a specific index of working memory, was the measure of interest. An alternation was defined as consecutive visits to four distinct arms in a row: for instance, ACDB and DABC but not ADBA or CBCA. The alternation rate was calculated as the total number of alternations divided by the total number of visited arms minus three. Re-entries into the same arm just exited were not counted, so that chance performance (poor memory) was an alternation rate of 22.2%. Mice with fewer than 15 arm entries were excluded from the analyses.

### Delayed non-matching to position (DNMTP).

Delayed conditional discrimination procedures such as DNMTP are used to assess short-term working memory in species ranging from worms to humans, and are sensitive to the types of impairments exhibited by Alzheimer patients and animal models of dementia [50] [51] [52]. In the present study mice were tested on a DNMTP task in hybrid operant chambers consisting of a touch-sensitive screen (iNexio LCD monitor; Lafayette Instruments, USA) directly coupled with a Med Associates operant chamber housed in a sound-attenuating cubicle. Each chamber was equipped with a food magazine, a house light, a tone generator, and a food pellet dispenser. The food receptacle (hopper) was located on one side of the chamber and the touch-screen on the opposite side. White squares were used as visual stimuli ( 5 × 5 cm), displayed on either the left or right side (or both) of the touch-screen, separated by 4 cm. Operant chamber outputs were controlled by graphical task design software (ABET II Touch; Lafayette Instruments, USA). 14-mg dustless precision food pellets (#F05684, Bio-Serv, USA) were used as reinforcers. Mice received one session per day, 5 or 6 days per week.

Initial training procedures were adapted from Leach et al. [53]. Food-deprived animals were gradually habituated to the environment and trained to retrieve a pellet from the hopper following the onset of a brief tone. When they retrieved 36 pellets in less than 60 min, an autoshaping procedure was initiated to train them to associate a stimulus (white square) with reinforcer delivery. Initially food pellets were delivered for a nose-poke response on the white square, or after 30 s in the absence of a response. In the next phase animals were required to nose-poke the displayed stimulus to obtain the reinforcer. A successful nose poke was followed by the illumination of the food well, a tone, and the delivery of a single food pellet. An inter-trial interval (ITI) of 15 s was introduced between the collection of the reinforcer and the start of the next trial. When performance was stable a mediating response was initiated, requiring a nose poke into the food well to initiate the trial and illuminate the stimulus. Finally, simple discrimination contingencies were introduced, during which a nose poke on the stimulus was required to initiate each trial (sample phase). A nose poke on the sample stimulus extinguished the sample stimulus and initiated the mediating phase. The mediating response illuminated the choice stimulus (choice phase). When the sample stimulus appeared on the left side of the screen, the choice stimulus appeared on the right, and vice versa. During pretraining, mice advanced to the next stage after completing 36 trials in less than 60 min, with at least 75% accuracy over two consecutive sessions. Non-Tg and 5xFAD groups took 21.1 (± 0.82) and 20.5 (± 0.95) sessions to reach criterion, respectively.

Following training, a DNMTP task was used to assess working memory. The DNMTP task was identical to the simple discrimination task except that a 1-s delay was interposed between offset of the choice stimulus and illumination of the food well for the intermediating response, and that both left and right choice stimuli were illuminated. Despite both being illuminated, only one response was deemed correct—a left nose-poke when the sample stimulus was on the right, and a right nose-poke when the sample stimulus was on the left (i.e., non-matching to position). For each session, mice remained in the chambers until they had performed a total of 54 trials or until 75 min had elapsed. A correct response was followed by the delivery of a food pellet and a tone. An incorrect response resulted in a time-out period (5 s) with the houselight turned on, followed by a 15-s inter-trial interval (ITI). Each incorrect trial was followed by a correction trial using the same non-matching spatial configuration as the incorrect trial. Correction trials continued until a correct response was emitted, but were not counted in the total number of trials completed. If the subject did not respond for 30 s (omission), both stimuli were extinguished and a time out initiated. The percentage of correct responses and omissions, along with response and hopper latencies were analyzed. Two months after AAV treatment, mice were again food-restricted and re-evaluated on the DNMTP task. All mice completed 30 acquisition sessions with a 1-s delay before AAV treatment, and 18 1-s re-acquisition sessions after treatment. The series of acquisition and re-acquistion sessions were each followed by a session using variable delays—1, 5, or 10 s, to generate delay functions.

### Histology and immunohistochemistry.

At 10- to 11-months old mice used for histology were given an intraperitoneal injection of Ketamine/Xylazine (92.25 mg/kg Zetamine^®^, VetOne, USA; 13.85 mg/kg AnaSed^®^, Akorn, USA), and perfused transcardially with ice cold saline, then with 4% paraformaldehyde (PFA). Brains were collected, postfixed in 4% PFA, and transferred solutions of increasing gradients of sucrose. 40-μm coronal sections were taken using a CM3050S cryostat (Leica, USA) and stored in antifreeze solution at −20° C. For choline acetyltransferase (ChAT) staining, 5–8 sections from regions anterior to Bregma (AP + 1.1 to + 0.8 mm) were selected for each animal [54]. These last coordinates span an optimal window to count ChAT-positive neurons in the medial septum and vertical limb of the diagonal band (MS/vDB; Ch1/2). All sections were first washed with phosphate buffered saline (PBS), then pre-treated with 70% formic acid (amyloid plaques) or 1% hydrogen peroxide (ChAT) for 5 min. After three PBS washes, sections were incubated for 48 hours at 4° C in primary antibody (Table 1) solution using 1% BSA and 0.3% Triton X-100 dissolved in PBS. For ChAT immunohistochemistry, following incubation of the primary antibody sections were washed thrice with PBS before a 24-hour long incubation period at 4° C in a biotinylated rabbit anti-goat secondary antibody. After additional PBS washing steps, all sections were incubated using an avidin/biotin-based peroxidase kit (Vectastain Elite; Vector laboratories, USA) for 3 hours at room temperature and further revealed with 3.3-diaminobenzidine (Sigma-Aldrich, USA). Sections were then mounted on gelatin-coated slides, dehydrated and covered with a cover slip. Images of the MS/vDB (ChAT immunostaining) were captured with a microscope equipped with a digital camera (Leica, USA). The open-source software Fiji (ImageJ) was used to quantify the average number of ChAT-positive neurons in the MS/vDB.

### Western Blotting and ELISA assays.

Mice used for neurochemistry were sacrificed under isoflurane anesthesia and brains rapidly removed. Hippocampal and frontal cortical tissues were dissected and immediately snap frozen in liquid nitrogen. For immunoblots, samples were homogenized in a RIPA buffer containing 25 mM Tris Hcl, 150 mM NaCl, 1% NP-40, 1% sodium deoxycholate, 0.1% SDS, and 2 mM EDTA (Thermo Fisher Scientific, Waltham, MA, USA). A protease/phosphatase inhibitor cocktail (HaltTM, dilution 1:100; Thermo Fisher Scientific) was added to the buffer to stabilize phosphorylated targets. After centrifugation at 14,000 rpm for 10 minutes at 4° C, supernatants were collected. For ELISA, hippocampal and cortical tissues were homogenized in 7 volumes of lysis buffer containing 120 mM NaCl, 50 mM Tris and protease/phosphatase inhibitor cocktails (Roche, Switzerland; Thermo Fisher Scientific). Protein lysates were centrifuged at 14,000 rpm for 15 minutes at 4° C and the supernatant (soluble Aβ fraction) was transferred to a different tube. The pellets were resuspended in 2% SDS and 1 μL of benzonase (Sigma-Aldrich, USA), sonicated, and centrifuged at 14,000 rpm for 15 minutes at 4° C. The corresponding supernatant (insoluble Aβ fraction) was transferred to a different tube. Homogenate concentrations were determined using a Pierce BCA protein assay (Thermo Fisher Scientific). Total, soluble, and insoluble Aβ40 and Aβ42 fractions were quantitated using commercially available kits (#27718 and #27719; IBL America, USA) according to the manufacturer’s instructions. For Western blots, proteins were resolved on precasted Any kD electrophoresis gels (Bio-Rad, USA), then transferred to PVDF membranes. After blocking for 1 h at room temperature in TBST with 5% nonfat dry milk, membranes were incubated over night at 4° C in TBST with 5% BSA, to which the primary antibody was added (antibodies used are listed in Table 1). Following washing steps in TBST, membranes were incubated for 1 h at room temperature with relevant HRP-conjugated secondary antibodies prepared in the antibody diluent. After final washing steps, immunoreactivity was detected using an ECL kit (SuperSignalTM West Pico or Femto; Thermo Fisher Scientific). Membranes were imaged with the Odyssey Fc imaging system (Li-Cor, USA). Signal intensity was directly quantified with the associated image processing software, Image Studio Lite.

### Data analysis.

Most behavioral, histological, and neurochemical data were analyzed using 2- or 3-way analysis of variance (ANOVA) or repeated-measures ANOVA (RMANOVA), with genotype, treatment, and gender as between-subjects variables. Follow-up comparisons were made using orthogonal contrasts. Neuronal culture groups were compared using one-way ANOVA followed by Dunnett’s tests. Categorical data were analyzed using χ^2^. Cross-maze data were analyzed using single-sample t-tests against chance (22.2%). Heteroscedastic data on the horizontal-beam task were square-root transformed before analysis. Time-series data were analyzed using hierarchical linear modeling, with time as a balanced, continuous repeated measure and subject as a nominal random factor nested within treatment, lesion, and gender. One outlier was removed from the synaptophysin assay for having unacceptably low GAPDH protein levels and expressing at 8.3 standard deviations above thed group mean. To protect against spurious Type I errors, *post hoc* tests were conducted on the Gender X Session interaction using Bonferroni-corrected t-tests. All statistical tests were two-tailed with α = 0.05.

## Results

**Figure 1:**
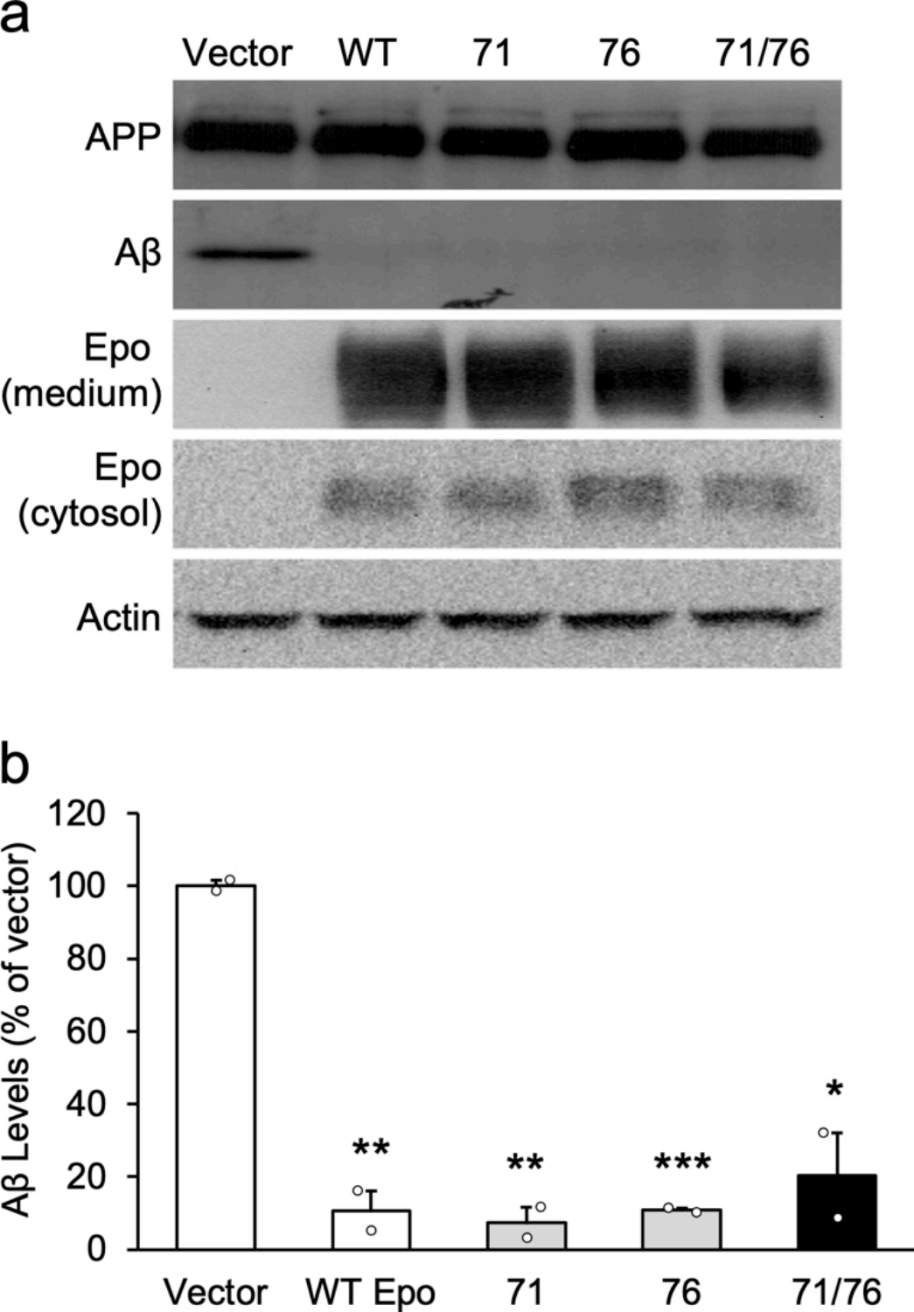
Wild-type and mutant Epo variants significantly reduce Aβ in 72a cells. 7PA2 cells were transfected with wild-type Epo or one of three Epo variants: R71S (71), R76E (76), or R71S/R76E (71/76). (**a**) Secreted Ab was precipitated with trichloroacetic acid and detected by immunoblotting, with actin as a loading control. (**b**) Quantitative analysis of the effects of Epo and its mutants on Ab secretion from two independent immunoblotting experiments. *p < .05; **p < .01; ***p < .001 vs. Vector; n = 2/group.

**Figure 2:**
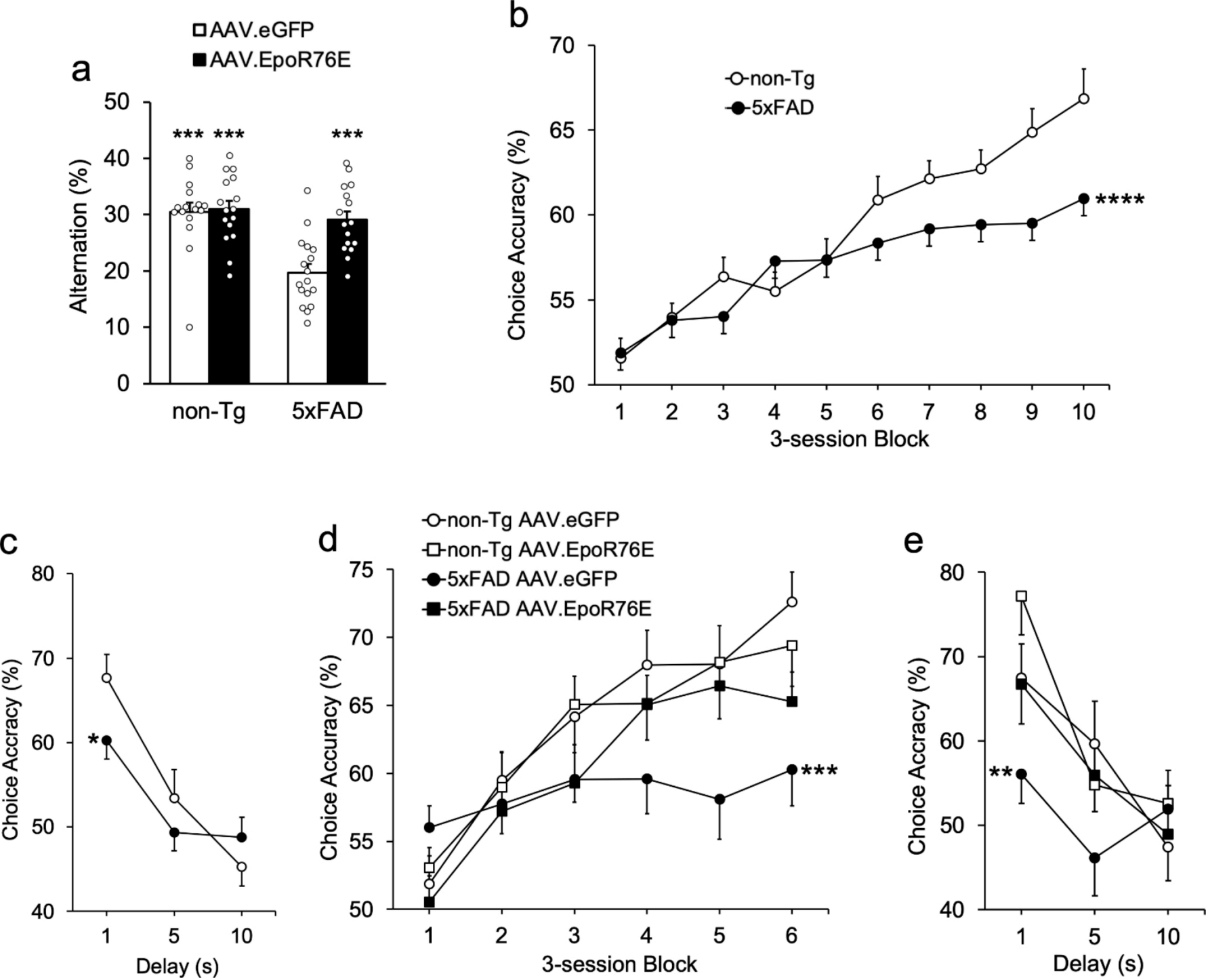
AAV.EpoR76E prevents short-term memory deficits in 5xFAD mice. (**a**) 5xFAD transgenics alternated at chance levels (22.2%) on the cross-maze alternation task 5 months after injection with the AAV.eGFP control vector. When injected with AAV.EpoR76E however, spatial memory was intact. (**b**) Around 4.5 months of age, 5xFAD transgenics learned to perform the DNMTP short-term memory task to proficiency, but performed significantly worse than non-Tg mice. (**c**) When challenged with longer delays, both groups showed characteristic memory decay functions, but transgenic mice were significantly worse. (**d**) Two months after treatment with AAV.eGFP or AAV.EpoR76E, mice were retrained on the DNMTP task. 5xFAD mice injected with AAV.eGFP were significantly impaired, but those injected with AAV.EpoR76E performed just as well as their non-Tg counterparts. (**e**) Post-treatment delay functions illustrate that transgenic mice injected with AAV.eGFP performed little better than chance on the 2-choice task, but those injected with AAV.EpoR76E performed as well as non-Tg mice. *p < .05; **p < .01; ***p < .0001 interaction vs. non-Tg (b,c) or non-Tg AAV.eGFP control (d,e). ***p < .001 vs. chance; n = 10–12/group (a,d,e) or 22–26/group (b,c).

7PA2 cultures were treated with AAV vectors containing either wild-type Epo, EpoR71S, EpoR76E, or EpoR71S/R76E. Aβ was extracted from the medium and analyzed by Western blot. AAV.EpoR76E significantly reduced Aβ expression by nearly 90%, and there was a significant overall effect on Aβ production [Fig. 1; F(4,5) = 41.3, p = .0005]. Follow-up tests showed that native Epo and all three AAV-driven Epo variants significantly reduced Aβ expression compared to vector alone [q’s > 10.6, p’s < .0009]. On the cross-maze test of spontaneous alternation, both non-Tg groups and the transgenics injected with AAV.EpoR76E alternated at rates significantly above chance [Fig. 2a; t’s > 4.6, p’s < .0002]. In contrast, 5xFAD mice injected with AAV.eGFP performed no better than chance, indicating poor spatial memory for arms previously visited [t(16) = 1.7, p = .941].

After training, mice completed 30 DNMTP sessions with a 1-s delay before AAV treatment. 5xFAD transgenics exhibited impaired learning of the DNMTP task (Fig. 2b), as indicated by a significant effect of genotype [F(1,61) = 4.3, p = .0416] and a significant Genotype X Block interaction [F(1,577) = 19.4, p < .0001]. In the next phase mice received a 54-trial session with variable delays of 1, 5, and 10 s to assess short-term working memory. Both groups performed best at the 1-s delay, and accuracy declined with increasing delay [Fig. 2c; F(1,92) = 40.4, p < .0001]. 5xFAD transgenics had significantly poorer working memory than non-Tg controls, indicated by a significant Genotype X Delay interaction [F(1,92) = 5.1, p = .0265]. Main and interaction effects of gender were not significant during either pre-training phase [F’s < 2.4, p’s > .127].

Mice started re-acquisition sessions on the DNMTP task 2 months after treatment, and most groups relearned within 18 sessions (Fig. 2d). There was a significant Gender X Session interaction that did not vary by genotype or treatment [not shown; F(1,217) = 4.6, p = .0325]. This reflected the tendency for female mice to perform better than males with increasing practice, although they did not differ significantly overall (61.6% vs. 61.7%) or on any of the 3-trial blocks (not shown; t’s < 1.9, p’s > .074). There was a significant main effect for genotype and significant genotype interactions with training block [F’s > 6.3, p’s < .017]. There was also a significant interaction between treatment and training block [F(1,217) = 4.0, p = .0465], and a significant Genotype X Treatment X Block interaction [F(1,217) = 14.3, p = .0002]. Follow-up mixed models showed that among mice treated with the AAV.eGFP control vector, 5xFAD transgenics performed significantly worse than non-Tg mice across sessions [F(1,108) = 31.4, p < .0001]. In contrast, transgenic mice receiving AAV.EpoR76E re-acquired the DNMTP contingencies just as well as their non-Tg counterparts [F(1,113) = .01, p = .924]. Another delay function was established after re-acquisition to examine the effect of AAV.EpoR76E on short-term working memory. There were significant main effects for both treatment and genotype [F’s > 8.1, p’s < .008]. Follow-up contrasts showed that transgenic mice treated with AAV.eGFP performed significantly worse than AAV.eGFP-treated controls [Fig. 2e; F(1,41) = 7.5, p = .0091]. In contrast, the choice accuracy of non-Tg and 5xFAD mice treated with AAV.EpoR76E did not significantly differ [F(1,41) = 3.0, p = .089]. There were no significant effects of gender or its interaction with any other factor [F’s < 4.0, p’s > .053].

Additional non-mnemonic data are collected in the DNMTP sessions that can be important for interpreting performance on the DNMTP task as well as other tasks, e.g., impaired or unusually slow behavior might be misinterpreted as poor memory. The last five sessions combined for each subject were used to evaluate these factors, i.e., sessions 26–30 pre-treatment and 14–18 post-treatment. There were two significant genotype differences pre-treatment, when mice were 5.5–6 months old (Table 2). All other effects were non-significant [F’s < 2.0, p’s > .163]. The number of correction trials per session was significantly higher in transgenics compared to non-Tg mice, consistent with their poor memory performance [F(1,62) = 5.3, p = .0247]. Hopper latency, the amount of time needed to collect the food pellet following a correct response, was significantly slower in 5xFAD mice [F(1,62) = 7.1, p = .0099]. Following treatment, at 8.5–9 months of age, there were significant main effects of genotype on five of the eight non-mnemonic factors [Table 3; F’s > 5.7, p’s < .022]. There were no other significant main or interaction effects [F’s < 4.1, p’s > .0503]. Follow-up contrasts showed that the correction trials and ITI responses per trial were significantly higher in 5xFAD transgenics of both treatment groups, compared to their respective non-Tg control [F’s > 4.4, p’s < .042]. Hopper latency, which was slower in 5xFAD mice before treatment, was still slower post-treatment but only significant in AAV.eGFP-treated transgenics [F(1,41) = 11.5, p = .0016]. In contrast to slower hopper latencies, sample latencies were faster in transgenics than controls, but again the difference was only significant in 5xFAD mice treated with AAV.eGFP [F(1,41) = 5.0, p = .0308]. Omissions are trials on which the subject failed to respond at all, and are considered a measure of poor performance. Interestingly, 5xFAD mice had fewer omissions before treatment, but not significantly so. After treatment the genotype difference was significant but only in transgenics treated with AAV.EpoR76E [F(1,41) = 6.2, p = .0171].

**Figure 3:**
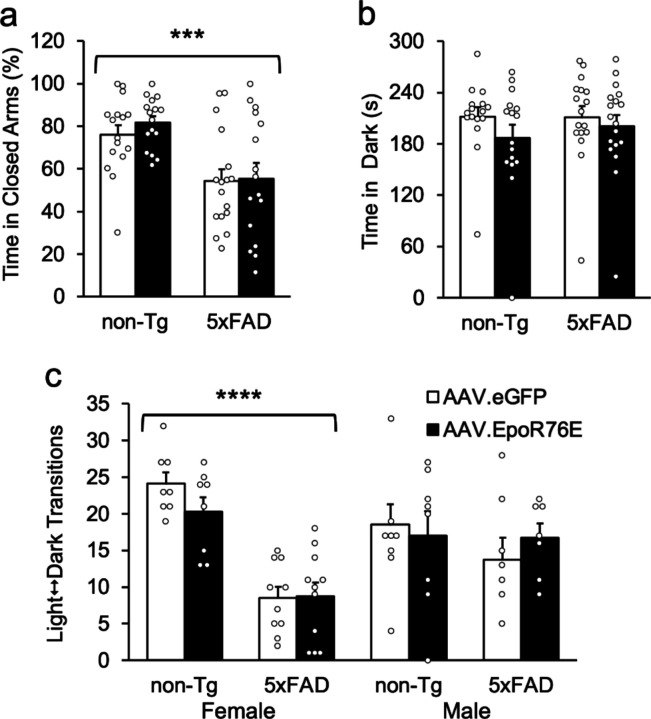
AAV.EpoR76E does not improve performance in the plus maze or light/dark tests in 5xFAD transgenics. (**a**) 5xFAD mice spent significantly less time than non-Tg controls in the closed arms of the elevated plus maze, but it did not differ by treatment. (**b,c**) Groups did not differ in the amount of time spent in the dark compartment on the light/dark test, but female transgenics had fewer transitions than non-Tg mice regardless of treatment status. Their male counterparts did not differ significantly by genotype. ***p < .001; ****p < .0001; n = 15–17/group.

It has been reported that 5xFAD transgenics spend less time in the closed arms of the elevated plus maze than controls, a behavior normally interpreted as reduced anxiety [55, 56]; however, this interpretation has been called into question [35]. Consistent with these studies, transgenic mice in the present study spent less time in the closed arms of the plus maze than non-Tg controls [Fig. 3a; F(1,57) = 20.5, p < .0001]. Closed-arm time did not differ by treatment or gender or their interactions [F’s < 3.0, p’s > .091]. On the light/dark test, groups did not differ by genotype, treatment, or gender in the time spent in the dark [Fig. 3b; F’s < 2.2, p’s > .144], but there were significant effects of genotype and its interaction with gender in the number of light-dark transitions [F’s > 14.3, p’s < .0005]. Transitions did not differ by treatment [F’s < 0.9, p’s > .35]. Follow-up contrasts showed that female 5xFAD mice had significantly fewer light-dark transitions than female non-Tg mice [Fig. 3c; F(1,59) = 38.9, p < .0001]; male mice did not differ [F(1,59) = 0.3, p = .594].

**Figure 4:**
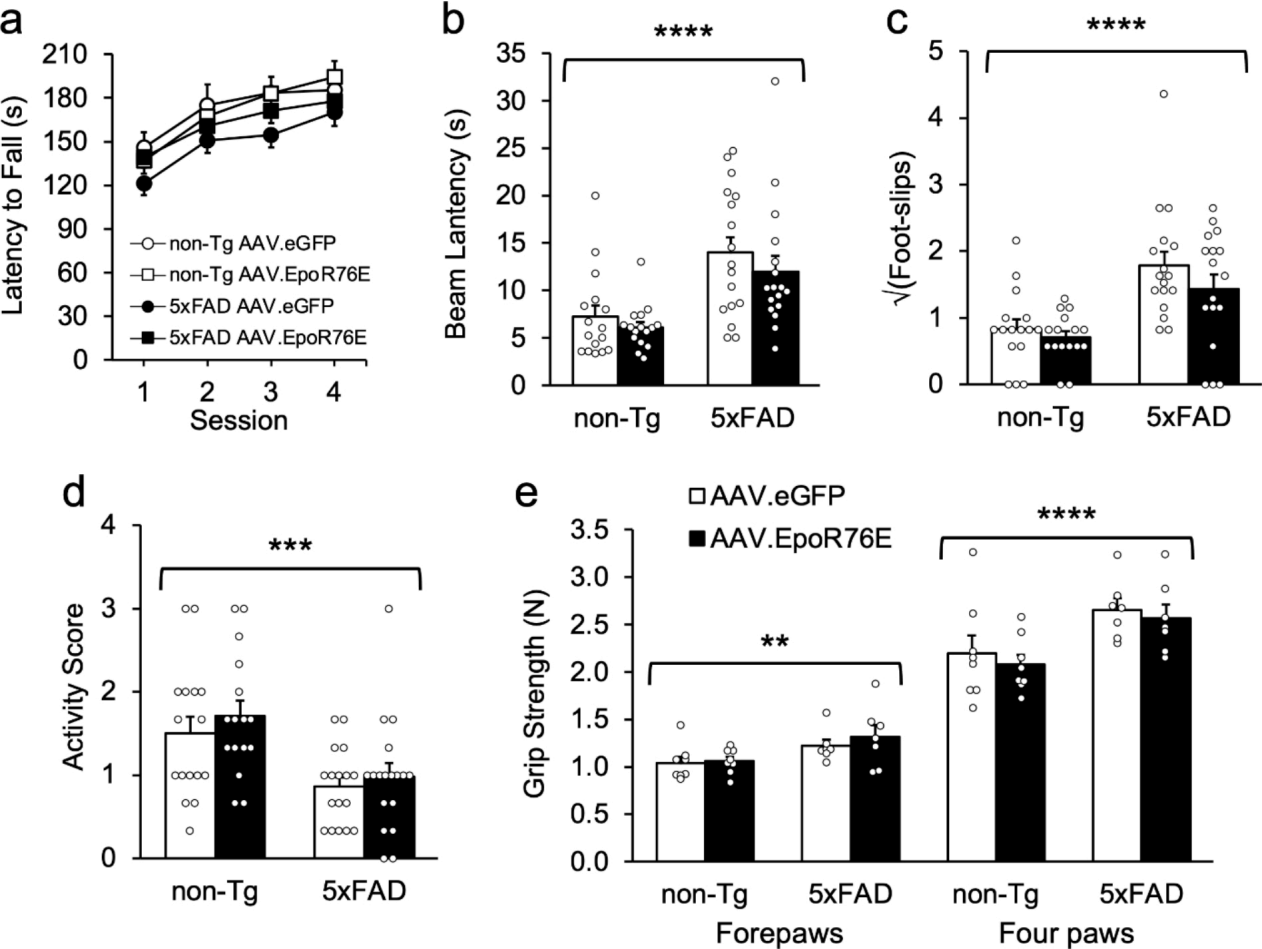
AAV.EpoR76E does not rescue motor deficits in 5xFAD transgenics. (**a**) There were no significant effects of genotype or treatment on the rotarod test of balance and coordination. (**b,c**) On the horizontal beam task transgenic mice took longer to cross and had more foot-slips than non-Tg controls, regardless of treatment. There was no gender effect on this task. (**d**) Latency to fall on the grid suspension task did not differ by genotype (not shown), but 5xFAD mice receiving either treatment were significantly less active on the grid suspension test than their non-Tg counterparts. (**e**) Male transgenic mice had significantly stronger grip strengths than non-transgenics, under both treatment conditions and when measured using forepaws only or all four paws. Female mice did not differ in grip strength under any condition (not shown). **p < .01; ***p < .001; ****p < .0001; n = 15–17/group.

On the rotorod task, there were no significant effects of genotype, treatment, gender, or their interaction, but there was a significant Genotype x Treatment x Session interaction [Fig. 4a; F(1,187) = 4.7, p = .0311]. Follow-up comparisons yielded no significant effect for treatment or genotype or their interactions with session [F’s < 3.9, p’s > .052]. There were significant effects of genotype on the horizontal beam task, indicating that transgenics had slower crossing latencies and more foot slips than non-Tg mice, regardless of genotype [Figs. 4b,c; F’s > 20.9, p’s < .0001]. There were no other significant main or interaction effects [F’s < 3.9; p’s > .055]. There was no significant main or interaction effect on latency to fall on the grid suspension test [F’s < 3.8, p’s > .056]. Nearly all mice in all groups were able to stay up the full 60 s. However, transgenics were significantly less active than non-Tg mice on the grid during the session [Fig. 4d; F(1,59) = 15.8, p = .0002]. There were no other significant main or interaction effects on activity score [F’s < 3.7, p’s > .061]. There were significant effects of genotype, gender, and their interaction with genotype on both measures of grip strength [F’s > 7.1, p’s < .0097]. Treatment effects were not significant [F’s < 2.5, p’s > .121]. Follow-up contrasts showed that among female mice there were no differences on any condition [data not shown; F’s < 1.0, p’s > .339]. In contrast, male transgenics held onto the grid significantly longer than male non-Tg mice on both measures [Fig. 4e; F’s > 11.0, p’s < .0016].

**Figure 5:**
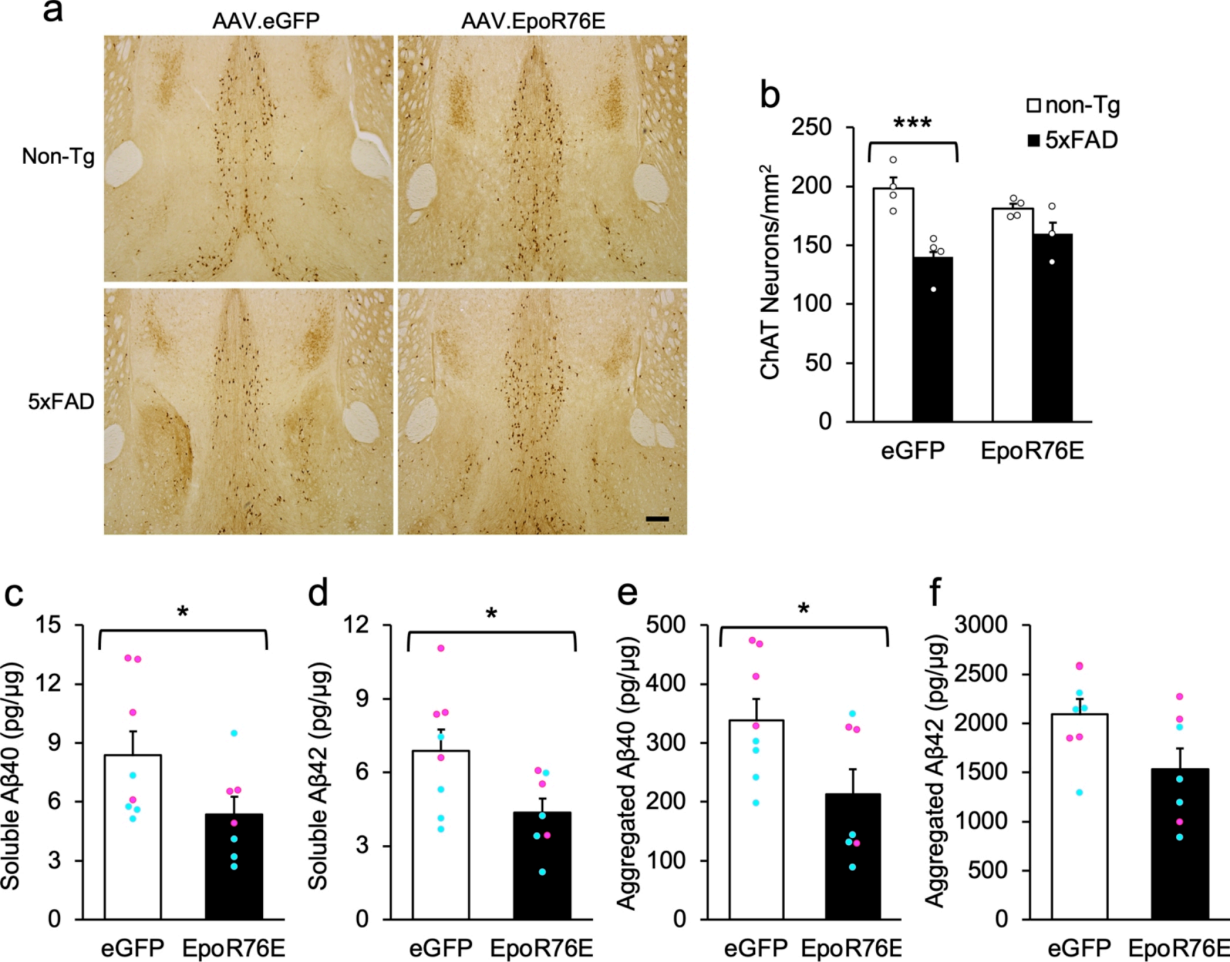
AAV.EpoR76E treatment is neuroprotective in the MS/vDB and decreases Aβ in 5xFAD transgenics. (**a**) Representative images of ChAT staining in the MS/vDB region; scale bar = 1 mm. (**b**) Quantification of ChAT-stained neurons showed a significant loss in 5xFAD transgenics injected with AAV.eGFP, but not in their AAV.EpoR76E-treated counterparts. (**c-e**) Soluble Aβ40 and Aβ42 and aggregated Aβ40 were significantly reduced in mixed cortical/hippocampal homogenates of 5xFAD mice treated with AAV.EpoR76E, compared to control transgenics injected with AAV.eGFP. Female mice (magenta circles) had significantly higher levels of all three Aβ species, regardless of treatment, compared to male mice (cyan circles). (**f**) Aβ42 did not significantly differ by treatment or gender. *p < .05; ***p < .001; n = 3–4/group (b) or 7–8 per group (c-f).

ChAT-positive cell counts in the MS/vDB differed significantly by genotype [F(1,7) = 23.0, p = .0020] and its interaction with treatment [Figs. 5a,b; F(1,7) = 7.6, p = .0280]. Follow-up contrasts showed that 5xFAD mice injected with AAV.eGFP had 29.3% fewer neurons than their non-Tg counterparts [p = .0008], but AAV.EpoR76E-treated groups did not differ [p = .215]. There was also a significant Genotype X Gender interaction in the counts of ChAT-stained neurons [not shown; F(1,7) = 6.4, p = .0390]. Follow-up tests showed that among 5xFAD mice, females had 15.9% more neurons per unit area than males [p = .0469], but non-Tg mice did not differ by gender [p = .3041].

Transgenic mice treated with AAV.EpoR76E had significantly lower levels of soluble Aβ40 and Aβ42, and aggregated Aβ40, compared to their AAV.eGFP-injected counterparts [Fig. 5c-e; F’s > 5.1, p’s < .045]. Aggregated Aβ42 was also lower (26.9%) in AAV.EpoR76E-treated mice, but the difference was not statistically significant [Fig. 5f; F(1,11) = 4.2, p = .065]. Consistent with previous ndings in APP-overexpressing mice and Alzheimer’s patients, there were also significant gender effects on Aβ species. specifically, female 5xFAD mice had significantly elevated levels of soluble Aβ40 and Aβ42, and aggregated Aβ40 compared to male transgenics, ranging from 56.5 to 61.6% higher [Figs. 5c-e; F’s > 5.3, p’s < .042]. Aggregated Aβ42 was also higher in females by 21.6%, but it was not statistically significant [Fig. 5f;F(1,13) = 1.6, p = .233]. Treatment x Gender interactions were not significant [F’s < 2.1, p’s > .180].

**Figure 6:**
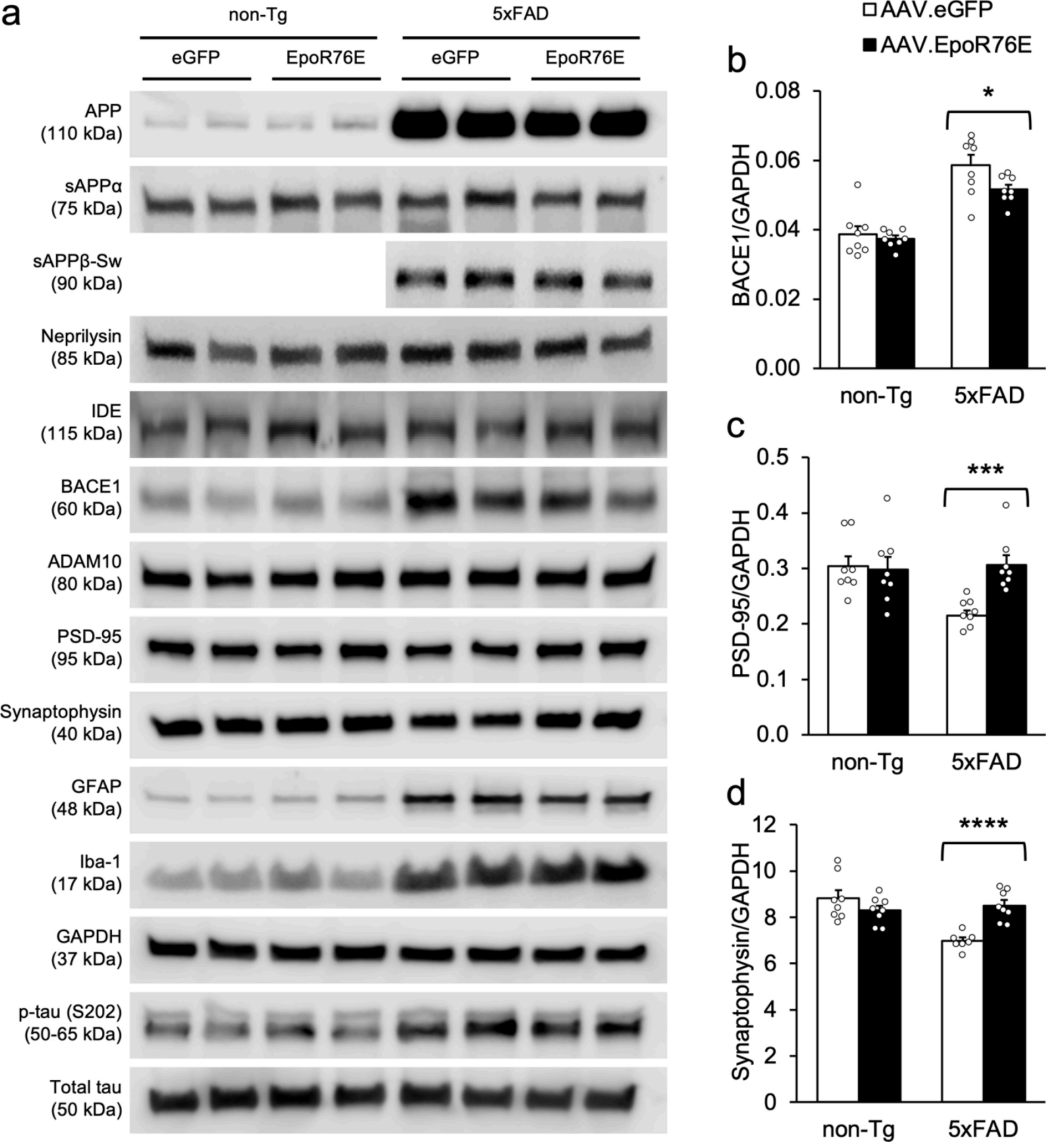
AAV.EpoR76E affects Aβ biosynthetic enzymes and prevents loss of synaptic markers. (**a**) Representative immunoblots from hippocampal homogenates. Total tau was used as a loading control for p-Tau (S202), and GAPDH for all others. (**b**) BACE1 was higher in transgenics than non-Tg mice, but significantly lower in transgenics injected with AAV.EpoR76E than their AAV.eGFP-treated counterparts. (**d,e**) The loss of synaptic proteins PSD-95 and synaptophysin typically observed in 5xFAD mice was evident in transgenics injected with AAV.eGFP, but not in those treated with AAV.EpoR76E. *p < .05; ***p < .001; ****p < .0001; n = 7–8/group.

We next examined proteins associated with APP and Aβ processing, synapse formation, neuroinflammation, etc. There was a significant genotype effect for APP, neprilysin, p-Tau (S202), Iba-1, and GFAP [Fig. 6a; F’s > 12.2, p’s < .009], but no difference between treatment groups or gender in either genotype [F’s < 2.1, p’s > .168]. There were no effects of genotype, treatment, gender, or their interactions on IDE or sAPPα [F’s < 3.1, p’s > .119]. There was a significant Treatment X Gender interaction for sAPPβ-Sw in 5xFAD mice [not shown; F(1,12) = 6.0, p = .0301]. Follow-up contrasts showed that female transgenics treated with AAV.EpoR76E had significantly lower levels of this cleavage product than their AAV.eGFP-treated counterparts [p = .0073], but male mice did not differ [p = .801]. There were significant Genotype X Gender and Genotype X Treatment X Gender interactions on ADAM10 [not shown; F’s > 5.3, p’s < .0489]. Follow-up ANOVAs showed a significant gender effect in 5xFAD transgenics only [F(1,4) = 10.4, p = .0322]. This reflected a 17.8% increase in ADAM10 in male AAV-EpoR76E-treated transgenics that was not significant [p = .054], and no difference among females [p = .789]. ADAM10 did not differ among non-Tg mice, either by treatment, gender, or their interaction [F’s < 3.2, p’s > .152]. There was a significant genotype difference in BACE1 [F(1,24) = 60.8, p < .0001]. Although transgenic BACE1 levels were higher overall, those treated with AAV.EpoR76E had significantly lower levels than their AAV.eGFP-treated counterparts [Fig. 6b; F(1,24) = 4.8, p = .0380]. Synaptic markers PSD-95 and synaptophysin showed similar profiles, namely significant main effects for genotype, treatment, and their interaction [F’s > 4.5, p’s < .0425]. Follow-up contrasts showed no differences among non-Tg mice [p’s > .054] but both proteins were significantly higher in 5xFAD transgenics treated with AAV.EpoR76E than their counterparts injected with AAV.eGFP [Figs. 6c,d;p’s < .0008]. There were significant Genotype X Gender interactions for both PSD-95 and synaptophysin [not shown; F’s > 6.9, p’s < .0147]. In both cases non-Tg females had higher protein levels than non-Tg males [p’s < .0086], but no differences in 5xFAD transgenics [p’s > .161]. There was also a Treatment X Gender interaction for synaptophysin [not shown; F(1,23) = 5.8, p = .0246], reflecting higher levels among AAV.eGFP-injected females than males [p = .0145] but no difference in AAV.EpoR76E-treated mice [p = .471].

## Discussion

We demonstrate here for the first time that a single peripheral administration of a recombinant AAV vector expressing EpoR76E has disease-modifying effects in a transgenic mouse model of Alzheimer’s disease. The 5xFAD model recapitulates most key aspects of Alzheimer pathology, including brain lesions in the form of amyloid plaques and hyperphosphorylated Tau protein, synaptic pathology, neuronal loss, and neuroinflammation [32, 57]. Although EpoR76E treatment did not restore all normal functions in 5xFAD mice, it protected against cognitive impairment, presumably by promoting the maintenance of synaptic clefts and reducing Aβ-associated toxicity, possibly by reducing expression of the β-secretase BACE1. Altogether, these effects were sufficient to prevent the severe memory impairments that developed in the 5xFAD transgenics treated with the control vector.

Gene therapy is a potentially powerful tool to treat patients with neurological disorders, and it is important to assess potential adverse effects [58, 59]. To date, few behavioral effects of the recombinant AAV-mediated EpoR76E variant have been reported in the literature [23, 29]. Consistent with Dhanushkodi et al. [31], we observed no significant changes in exploratory locomotor activity, sensorimotor function, or anxiety-related behavior in the present paper (Fig. 4), allowing us to conclude that systemic AAV.EpoR76E itself had no overt adverse effects [60]. However, the modified Epo vector treatment did not improve the motor impairments typically seen in aged 5xFAD transgenics, or alter performance on the elevated plus or light/dark tests. A number of papers have shown that 5xFAD transgenics spend less time in closed arms of the elevated plus maze [35, 55, 56]. Although this is routinely interpreted as decreased anxiety, in a previous paper we showed that the 5xFAD transgenics were actively avoiding closed arms due to vibrissal hypersensitivity secondary to degeneration of inhibitory interneurons in the barrel field cortex [35]. When their whiskers were snipped they spent as much time in the closed and open arms as the non-Tg mice. Mice in that paper performed normally on all other tests of anxiety, including the light/dark test. Similarly, 5xFAD and non-Tg mice spent equivalent amounts of time in the dark on the light/dark test in the present paper, indicating normal anxiety (Fig. 3b). Although the number of light/dark transitions is sometimes considered a secondary index of anxiety, it is hard to make that interpretation without a concomitant change in time spent in the dark. Indeed, the reduced transitions in female transgenics in the present paper (Fig. 3c) may also be attributable to decreased locomotion or even to vibrissal hypersensitivity given that the opening between light and dark chambers was even smaller (5 cm) than the distance between walls of the closed arms of the plus maze (6 cm). Considering the sum of the data, we cannot conclude that the 5xFAD transgenics’ abnormal performance is attributable to decreased anxiety in the elevated plus maze or increased anxiety in the light/dark box.

With respect to cognition, wild-type Epo and derivative treatments are widely recognized to alleviate memory impairments in experimental models of Alzheimer’s disease and aging [61–64]. Consistent with these studies, AAV.EpoR76E reversed the cognitive decline usually observed in 5xFAD mice in two memory tasks in the present study, both of which rely on active engagement of hippocampal regions [65–67]. The DNMTP data are particularly powerful for several reasons. First, the task requires the subject to make a choice (as does the cross maze), rather than inferring memory from running or swimming. Thus mild-to moderate motor problems are controlled for, as the sensorimotor requirements for correct and incorrect responses are identical. Second, proficient performance at the shortest (1-s) delay affords additional controls, as we can be certain that subjects understand the rules, are motivated, and possess the sensorimotor capacity to complete the task. Third, the DNMTP task is directly analogous to the short-term working-memory tasks that are impaired in Alzheimer’s disease. Indeed, laboratory and clinical tests for short-term working memory in Alzheimer patients and those with mild cognitive impairment (MCI) often use variants of delayed conditional discrimination (DCD) tests such as DNMTP or delayed matching to sample (DMTS; [50, 68–74]). Fourth, it provides a powerful model with which to track the progression of dementia. The delay functions illustrate this progression. At the younger age transgenics performed nearly as well as non-Tg mice, showing the decrement in accuracy with increasing memory demand characteristic of short-term working memory forgetting curves (Fig. 2b). At the older age the deficit was worse in AAV.eGFP-treated transgenics, performing at roughly chance levels even at the shortest delay (Fig. 2d). This resembles the delay functions reported in Alzheimer’s patients. For example, both DNMTS and delayed matching-to-position (DMTP) variants of DCD tests can discriminate early- and late-stage Alzheimer’s patients with delay functions similar to the ones described in Fig. 2. Patients early in the disease or with MCI perform well at short delays, indicating good sustained attention, motivation, and understanding of the rules of the task, and accuracy decreases with increased delay. At later stages there are significant drops in the y-axis (shortest delay), indicating non-mnemonic processes are probably affecting performance [75–79].

In 5xFAD mice and other transgenic models of Alzheimer’s disease, overexpression of APP is accompanied by physiological and structural changes in the hippocampus, leading to the deterioration of cognitive abilities and decreased synaptic integrity [80, 81]. In the present study, hippocampal levels of presynaptic protein synaptophysin and postsynaptic scaffold protein PSD-95 fell by 18 and 27%, respectively, in transgenic mice receiving the AAV.eGFP control vector. Levels of both proteins were restored in 5xFAD animals following AAV.EpoR76E therapy, suggesting a potential role for EpoR76E in modulating synaptic plasticity. It is noteworthy that other studies using native Epo or its variants also reported improved synaptic integrity and memory [13, 82, 83] [84]. For instance, intraperitoneal Epo prevented memory deficits induced by bilateral infusion of Aβ25–35 in rats by concomitantly enhancing long-term potentiation and reducing paired-pulse ratio, an index of neurotransmitter release probability [85].

Synaptic failure also reflects the inability of neurons to cope with the cumulative toxicity of soluble Aβ oligomers in Alzheimer’s disease [86]. ln accordance with preliminary studies using Epo or its derivatives in Alzheimer models [13, 61, 87, 88], AAV.EpoR76E treatment in the present paper significantly decreased soluble and aggregated Aβ in mixed hippocampal-cortical homogenates. APP proteolysis is primarily triggered by α-secretases of the ADAM family (non-amyloidogenic pathway) or the major β-secretase BACE1 (amyloidogenic pathway), the latter leading to the misfolding of Aβ peptide and subsequent deposition of neuritic plaques. In the present study BACE1 was significantly reduced in 5xFAD mice injected with AAV.EpoR76E, compared to transgenics given the control vector. In addition, ADAM10 was non-significantly increased ~ 18% in male transgenics receiving the EpoR76E treatment. Although these effects are relatively small, they may have been sufficient to result in the modest reductions in Aβ that we observed in EpoR76E-treated transgenics, particularly since injections were given at an age (5.5–6 months) when 5xFAD mice already have substantial plaque formation in the cortex and hippocampus. Notably, soluble Aβ42 was reduced 36.5% in EpoR76E-treated transgenics compared to their eGFP-treated counterparts. Small soluble Aβ42 oligomers reduce synaptic proteins and are associated with more severe neurodegeneration and memory loss in Alzheimer’s disease and experimental models [89–92,93 {Shankar, 2007 #6586,94].

The reduction in soluble Aβ may have protected the MS/vDB neurons in the present study. Cholinergic innervation of the brain is altered early in Alzheimer’s patients, primarily due to neurodegenerative processes occurring in the septo-hippocampal pathway. Although most APP-overexpressing transgenics do not exhibit neurodegeneration, 5xFAD mice show robust neuronal loss in the MS/vDB and other regions [32, 35, 55]. The MS/vDB provides cholinergic input to the hippocampus and entorhinal cortex, and our quantification of ChAT-positive neurons showed that AAV.EpoR76E was neuroprotective. Importantly, a critical analysis of region-specific lesions in models of Alzheimer’s disease concluded that insults to the medial septal area best modeled the progression of short-term memory deficits observed in Alzheimer’s patients and modeled by DCD tasks such as the DNMTP [50].

The data presented herein demonstrate that a single injection of a vector that produces a modified Epo variant can protect neurons, preserve synaptic integrity, and prevent cognitive decline in a model of Alzheimer’s disease. This is consistent with our previous study showing significant (~ 50%) neuroprotection in the MPTP model of Parkinson’s disease following a single intramuscular injection of AAV.EpoR76E, as well as studies showing that AAV.EpoR76E protects against neurodegeneration in models of glaucoma and retinal degeneration [23, 31, 36, 95]. Given that only a single injection was given and beneficial effects observed up to 5 months later, this suggests that a genetic therapy propagating a non-hematopoietic Epo variant such as EpoR76E may be a viable treatment option for Alzheimer’s disease and other neurodegenerative conditions.

## Supplementary Material

Supplement 1 Table 1 to 3 are available in the Supplementary Files section.
