# Supplementary material for: Intramuscular delivery of recombinant AAV expressing EpoR76E improves memory and is neuroprotective in 5xFAD transgenics
Source: Res Sq. 2025 Apr 18:rs.3.rs-6465973. Preprint. [Version 1] doi: 10.21203/rs.3.rs-6465973/v1 (PMC12047997; doi:10.21203/rs.3.rs-6465973/v1)
Supplement: Supplement 1 — Table 1 to 3 are available in the Supplementary Files section. [file NIHPPrs6465973v1-supplement-1.pdf]

Supplementary Files

This is a list of supplementary files associated with this preprint. Click to download.

- 5xFADEpoTables.pdf
